# Health-Related Quality of Life and Coping Strategies in a Cohort Study of Highly Active Antiretroviral Therapy Naïve Patients Adherence

**DOI:** 10.1155/2022/8341638

**Published:** 2022-02-09

**Authors:** Patricia de Oliveira França, Lorena Rocha Ayres, Lúcia Helena Pimassoni, Crispim Cerutti Junior

**Affiliations:** ^1^Post-Graduate Program of Infectious Diseases, Federal University of Espirito Santo, Vitória, Espirito Santo, Brazil; ^2^Department of Pharmaceutical Sciences, Federal University of Espirito Santo, Vitória, Espirito Santo, Brazil; ^3^School of Medicine, Escola Superior de Ciências da Santa Casa de Misericórdia de Vitória, Vitoria, Espirito Santo, Brazil

## Abstract

**Objective:**

The main objective of this study was to describe the perceived quality of life (QoL) in patients living with AIDS (PLWA) and their chosen coping strategies in a cohort of individuals managed with HAART.

**Methods:**

This is a prospective cohort study conducted at the Medication Dispensing Unit of a university hospital (MDU-UH) located in southeastern Brazil. Study population comprised HIV/AIDS patients starting antiretroviral treatment at MDU. The final sample comprised 99 participants. Patients were followed up for 24 months from their recruitment. We used a face-to-face questionnaire to determine sociodemographic and behavioural variables. Quality of life (QoL) and coping strategies (CS) were measured through validated instruments.

**Results:**

Regarding the QoL dimensions, the general perception of QoL among these participants was considered good. Regarding CS, the adherent patients scored higher than the nonadherents.

**Conclusions:**

The present study revealed that the perceived QoL can be maintained in individuals treated for HIV/AIDS. There is an association between high score of coping strategies and adherence to HAART.

## 1. Introduction

The improvement in the fight against HIV/AIDS can be attributed to the widespread use of highly active antiretroviral therapy (HAART) known as an effective HIV inhibitory medication therapy. HAART converted HIV infection from potentially fatal to the status quo of chronic disease, reducing the frequency of morbid episodes and hospitalizations, while also increasing the patient's survival [1, 2].

However, the medication's toxicity level ranges from moderate to severe and they must be taken regularly throughout the patient's life, whereas therapy discontinuation leads to resistance by the virus and an increase in its quantity in the individual's body [3–5]. Therefore, it is necessary to understand how HAART impacts on the quality of life (QoL) of people living with HIV/AIDS (PLWHA) [6], since this treatment interferes not only with the individual's physiological condition but also with their social and cultural aspects [7, 8].

Commitment in the care process is a critical step for PLWHA newly initiated on HAART. Albeit effective, these drugs are not sufficient for the full and effective maintenance of viral suppression if the patient does not effectively take them continuously and adequately, a practice that is improbable in a scenario of low perceived QoL [9].

Although bioclinical factors are important for antiretroviral therapy adherence, behavioural and social aspects of PLWHA must be considered. The therapeutic outcome in AIDS is not only a matter of pathogen versus host, development of new antiretroviral drugs, or adverse reactions; it is also a matter of cultural differences, social inequalities, poverty, stigmatization, and metaphorization of the disease [10–12].

The complexity of the relationship between HIV, HAART, and QoL lies in the fact that while medication is necessary for viral suppression and to improve patients' quality of life in short and medium term, extended use of ART can lead to several adverse events that decrease QoL [13]. In addition, the way the disease installs in the individuals life during the sickness process leads to the redefinition of things around them, whereas PLWHA adopt different strategies to cope with negative feelings such as anger, denial, and depression. These coping strategies are understood as cognitive and behavioural efforts to tackle stressful and resource demanding events [14], which are important to influence QoL psychosocial aspects and to support HIV-infected continuous treatment [15].

There are few studies on the impact of HAART on the QoL of PLWHA in patients initiating antiretroviral therapy and, above all, on the influence of QoL and coping strategies on adherence to antiretroviral medication [13, 16, 17]. Previous surveys have explored experienced patients to the detriment of those newly initiated on HAART; that is, understanding the impact of psychosocial factors on these patients will provide subsidies for improvement in current adherence strategies. Therefore, the main objective of this study is to determine the perceived QoL and coping strategies in naïve patients on HAART and pharmaceutical assistance.

## 2. Methods

### 2.1. Participants and Procedures

We conducted a prospective cohort study at the medication dispensing unit (MDU) of a university hospital (UH) located in southeastern Brazil. Patients were followed up for 24 months from their recruitment. The inclusion criteria were being older than 18 years, having started HAART in the study period at MDU, and being seen at the university hospital outpatient unit. We excluded from the sample patients who attended private clinics, inmates, and those incapable of fulfilling the proposed questionnaires or other instruments due to cognitive disablement. All eligible patients were invited to participate in the study. There were six dropouts at sample (*n* = 109) (i.e., two abandoned the treatment in the first three months, and eight lost the follow-up after 12 months of treatment). The final sample comprised 99 participants.

First, the data collection started with a face-to-face questionnaire applied before the start of HAART to assess patients' sociodemographic and behavioural aspects. The laboratory data (e.g., HIV viral load and CD4 count) was obtained from the laboratory test control system (SISCEL) and clinical information (i.e., initial clinical condition, whether asymptomatic or not, and existence of opportunistic infections) obtained both directly and from the patient's medical record. The inclusion of asymptomatic patients was possible because the outpatient unit is a reference for providing health care to HIV-infected persons, receiving referrals from the entire state. Thus, patients screened for HIV infection during surgical procedures, blood donation, prenatal care, or treatment for sexually transmitted infections are frequently referred to the unit.

Second, QoL was measured before the start of HAART. Both QoL and coping strategies (CS) were measured 12 months after the beginning of treatment using a translated and validated full version of WHOQOL-HIV BREF [17] and the Brazilian version of the ways of coping scale (WCS) [18]. Third, a replication of the second step was done 24 months after initiation of the HAART.

The WHOQOL-HIV BREF contains 31 items divided into six domains: physical (D1), psychological (D2), level of independence (D3), social relationships (D4), environment (D5), and spirituality/religion/personal beliefs (D6). Participants selected their responses in a 5-point Likert scale, whereas high scores mean better perceived quality of life. The WCS consists of 45 items subdivided into four factors: problem-focused coping (PFC), emotion-focused coping (EFC), search for religious practices/fanciful thinking (SRP), and search for social support (SSS). Participants selected their responses in a 5-point Likert scale ranging from (1) never to (5) always. Overall scores for each strategy were obtained by averaging the scores of their respective items. Higher values indicate greater use of a certain coping strategy. Patients' HAART adherence was measured through the Brief Medication Questionnaire. The BMQ is divided into three groups of questions, called regime, belief, and recall, to be completed by the patient. The “regimen” group starts by asking the patient to list the medications they take by name, how many pills they take at a time, and so on. It is related to the patient's knowledge of their treatment, in addition to obtaining the account of how this medication was taken. Next, the “belief” group seeks to evaluate whether the patient believes that the treatment is indeed working and addresses the patient's concerns or doubts regarding their treatment, as well as whether it is generating any discomfort to the user. Finally, the “recall” group analyzes whether the patient has any problem remembering to take the medication correctly. For each answer that is not in conformity, or that is different from what is expected, the patient is evaluated with a score that is added up, establishing a score that classifies the patient in adherents or nonadherents [19]. The BMQ combined with pharmacy medication dispensing profile records allows for a better estimation of patients' monthly missing doses. Those who have missed 10% were considered nonadherent [20].

### 2.2. Ethical Considerations

Patients who agreed on taking part in the study signed an informed consent form. This study was approved by the Research Ethics Committee of the Federal University of Espirito Santo, Health Sciences Center, under the protocol number 2008197.

### 2.3. Statistical Analysis

Background variables such as demographic, behavioural, clinical, and laboratory variables were presented as frequencies (n) and percentages (%). Differences in participants' QoL and CS score factors between the baseline and follow-up were assessed using the Wilcoxon Test.

The comparison of quality of life and coping strategies in the patient's adherence profile was assessed using the Student's t-test for independent samples or the nonparametric Mann-Whitney test in case of normality not detected by the Kolmogorov-Smirnov test. The correlations between the scores of each factor of the ways of coping scale and the scores of each domain of the WHOQOL-HIV BREF questionnaire were assessed using the nonparametric Spearman test.

To identify the factors that contributed to nonadherence in the group studied after two years of follow-up, all independent variables measured that had significance at the level of *p* < 0.2 in the bivariate analysis (Chi-square or Fisher's Exact Test) were entered into the multivariate logistic regression backward stepwise model. The Hosmer-Lemeshow test was performed to assess model acceptance. The results were presented as adjusted odds ratio (aj. OR).

## 3. Results

### 3.1. Background Variables

The majority were male (76.1%), 57.8% were nonheterosexual, 61.5% used alcohol, 78.9% did not use drugs, 81.7% had a viral load value over 1000 copies/ml, and 68.8% had more than 350 TCD4 cells/mm^3^. Nearly all participants started their treatment with a therapeutic regimen based on DTG/TDF/3TC (dolutegravir/tenofovir disoproxil fumarate/lamivudine). Detailed characteristics of the included subjects are reported elsewhere (França et al. forthcoming) and in Table 1.

### 3.2. Quality of Life

After 24 months of follow-up, participants reached higher scores in the physical QoL domain (mean (M) = 16.7; standard deviation (SD) = 2.7; median (Md) = 18.0; interquartile range (IQR): 15.0–19.0) and lower values in social relationships domain (*M* = 14.9; DP = 3.3; Md = 16.0; IQR: 13.0–17.0). Except for the social relations QoL domain, all the other domains had an increase in their scores, comparing the beginning of treatment and 12 and 24 months of follow-up (*p* < 0.05). The general perception of QoL among these participants was considered good (*M* = 15.6; SD = 2.1; Md = 15.9; IQR: 14.5–17.2), with a significant increase in total quality of life scores at 12 and 24 months of follow-up (*p* < 0.001) compared with the beginning of treatment (Table 2).

### 3.3. Coping Strategies

Among the coping strategies, search for religious practices/fanciful thinking (*M* = 3.8; SD = 0.8; Md = 3.8; IQR: 3.2–4.4) and emotion-focused (*M* = 2.2; SD = 0.7; Md = 2.1; IQR: 1.7–2.6) stood out as the most and least used strategies, respectively, by the participants after 24 months using antiretroviral therapy (ART). Comparing the coping strategies after 12 and 24 months of follow-up, only the search strategy for religious practices/fanciful thinking showed a significant increase in the measured scores (Table 3).

### 3.4. Association between Coping Strategies and QoL

According to Table 4, after 24 months of follow-up, the strongest correlations between QoL domains and WCS factors (Spearman Rho >0.4; *p* < 0.01) occurred negatively between emotion-focused coping strategy and QoL domain D2 (Spearman Rho = −0.413; *p* ≤ 0.001) and between emotion-focused coping strategy and QoL (Spearman Rho = −0.475; *p* ≤ 0.001).

### 3.5. Association between Coping Strategies, QoL, and Adherence

The median score for the problem-focused strategies and search for religious practices were the highest. However, they were not statistically associated. Coping strategies were associated with adherence on the search for social support factor (*p*=0.021) (Table 5). For this strategy, the adherents' patients scored higher than the nonadherents. Regarding the QoL dimensions, the general perception of QoL among these participants, whether adherent or nonadherent, was considered good. The higher score was obtained for the D1 domain (Md = 18.0; IQR: 15.0–19.0) (Table 2), although there was no statistically significant association between QoL dimensions and adherence (Table 5).

### 3.6. Adherence to ART

The independent variables that remained in the final logistic regression model related to the factors of the WHOQOL-HIV BREF and the WCS were, respectively, the physical domain and the spirituality domain and the social support-seeking factor of the WCS. The increase of the spirituality domain score of the WHOQOL-HIV BREF represents risk of nonadherence in 1.4 times for each increased unit (adjusted OR = 1.411; 95% CI: 1.411–1.057). In turn, the increase of the score in the social support-seeking factor of the WCS represented protection against nonadherence; that is, for each unit increased in the score of the factor, the chance of adherence increased by 75.7% (adjusted OR = 0.243; 95% CI: 0.086–0.692) (Table 6).

## 4. Discussion

The evaluation of the participants revealed a good and statistically significant general perception of QoL by these individuals throughout the 24 month period. When evaluating QoL measurements between different health conditions (i.e., patients with good vs. poor health), it is expected that those with higher levels of illnesses report lower QoL scores [21].

Across all possible QoL domains, participants scored higher on physical and level of independence, a result consistent with the studied sample, since 68.8% of the participants started treatment asymptomatic.

The social relations QoL domain did not vary over time (*p* > 0.05). This domain has facets related to personal relationships, social support, social activity, and social inclusion, assessing aspects related to acceptance in society, satisfaction with sex life, personal relationships, and support from friends. This is perfectly expected considering that HIV is still stigmatized and excluding disease, which limits the social integration of those affected [22, 23].

At the end of two years of follow-up, 80.8% of them were adherent, reinforcing the role of adherence to HAART in viral suppression, maintenance of adequate clinical conditions, and improvement of quality of life of PLWHA. We attribute a significant increase in the total quality of life scores at 24 months of follow-up to the maintenance of virological suppression by the HAART, which allows improvement of physical condition enabling to carry out daily activities.

At the end of the two year period, there was a significant increase in search for religious practices without altering the frequency of other coping strategies. Religious and spiritual practices are one of the most used strategies to manage the anguish and stress related to HIV-positive status [15]. Religious involvement can be part of the individual's life without relation to situations or conditions of experienced stress. On the other hand, in coping strategies, the individual would use religion as a tool to deal with the stress source [24].

When using religion as a coping strategy, people can adopt a collaborative approach that consists of working together with their God to solve the problem, placing their expectations of resolution entirely in the capacities of a given deity [25]. In the case of HIV patients, they could abandon treatment with antiretroviral drugs or not adhere to them in case of using this coping strategy. In our study, there was no statistically significant association between SRP and adherence, demonstrating that, in this situation, it did not interfere positively or negatively in the medication adherence. Accordingly, the increase in the WHOQOL-HIV BREF score in the spirituality domain represented an increased risk of nonadherence, suggesting a negative impact of this behaviour for the group under study. In contrast, the coping strategy search for social support of the WCS favored adherence. Individual behavioural aspects may influence adherence to ART, including their religious involvement. Religious beliefs may have positive effects on adherence processes [26, 27]. However, there are also negative impacts, mainly associated with the stigmatized view of HIV carriers and the indoctrination of some religions to preach healing by divine power in denying the use of antiretroviral drugs [28, 29].

The emotion-focused coping strategy had a negative correlation with the D2 domain of QoL, representing agreement with the scores obtained in the evaluation of the study group in the 24 months of follow-up, since the focus on emotion was the least used strategy by these individuals. Moreover, we noted an increase in the measured scores of D2 comparing the measurements at the beginning with those after one and two years of treatment. Emotion-focused coping is an individual response that focuses on managing emotional responses to a stressful event [24], encompassing cognitive and behavioural strategies that can fulfill an anodyne function in coping or result in distancing from the stressor [18].

The responses of the WCS social support factor have in common the fact that they involve another person in their emission, meaning they are social interactions, which require the performance of another individual, with his support network [14]. Previous studies pointed out that the lack of social support from relatives, friends, and partners is a relevant factor to increase depression among PLWHA resulting in lower adherence to ART [30].

In the process of adherence to antiretroviral therapy and its social representations for people living with HIV/AIDS, the family represents a support network of extreme importance for the continuity of adherence to drug treatment, constituting an agent of support to the process. Hence, nonadherence is an event determined by several factors, given their relationship with the person under treatment, the disease, the therapy, the health services, and social support [31].

Indeed, adverse reactions to ARVs are a challenge for not only PLWHA adherence but also their socioeconomic status [32–35].

Several systematic reviews addressing barriers to ART adherence in developed and developing countries have shown that fear of disclosing HIV-positive status, suspicion of being on treatment, and desire to avoid taking medications in public places, among others, negatively affect adherence [33]. However, “simply forgetting” has also been demonstrated as the most common reason for nonadherence to ARVs [36–40].

We recognize that the study had some limitations. The first was the small sample size, which makes difficult to detect differences between the different categories. Therefore, some associations did not remain after 24 months of follow-up. The second is linked to the study scenario, conducted in real-life conditions for PLWHA. Even though this is advantageous because it reliably portrays the conduct of the treatment, it prevents stricter control over all variables, as in controlled clinical studies.

Despite the limitations, the study succeeded in revealing the religious beliefs' negative influence on ART adherence process as well as the importance of the social support on the HIV care cascade.

## 5. Conclusion

The participants revealed a good and statistically significant general perception of QoL. In general, coping strategies and medication adherence were not associated except for SSS.

The increase in the WHOQOL-HIV BREF score in the spirituality domain represented an increased risk of nonadherence, suggesting a negative impact of this behaviour. In contrast, the coping strategy search for social support of the WCS favored adherence in the group studied.

This study reinforces the importance of the influence of the social component in the process of adherence to ART. Thus, a multidisciplinary look at the individual newly initiated on HIV treatment for the continued maintenance of the antiretroviral treatment is clearly needed. It is essential to empower the patient, making them conscious of their role in the treatment process. If there is no cure for their disease, the patient must become aware that there is control over its progression. In this aspect, the act of dispensing the antiretroviral medication must be surrounded by care, especially by the pharmacist. Our results provide evidence to the health authorities responsible for policies regarding HIV infection care to emphasize the need for multidisciplinary teams, pharmacist empowerment, and valorization of strategies privileging self-caring and social support.

## Figures and Tables

**Table 1:** Participants' sociodemographic, behavior, clinical, and laboratory characteristics (*n* = 109).

Participant variables	Number of participants	
	*n*	(%)
Age		
≤35	36	(33.0)
>35	73	(67.0)
Gender		
Male	83	(76.1)
Female	26	(23.9)
Skin colour/ethnicity		
White	37	(33.9)
Brown	51	(46.8)
Black	21	(19.3)
Marital status		
Married/common law marriage	21	(19.3)
Single	88	(80.7)
Schooling (years)		
1 to 8	36	(33.0)
9 to 12	36	(33.0)
More than 12	37	(34.0)
Monthly income †		
<3 minimum wage	92	(84.4)
≥3 minimum wage	17	(15.6)
Employment status		
Employed	93	(85.3)
Unemployed	16	(14.7)
Sexual orientation		
Heterosexual	46	(42.2)
Nonheterosexual	63	(57.8)
Alcohol use		
Yes	67	(61.5)
No	42	(38.5)
Illicit drug use		
Yes	23	(21.1)
No	86	(78.9)
Cigarette smoking		
Yes	43	(39.4)
No	66	(60.6)
Mode of transmission		
Sexual intercourse	73	(67.0)
Unknown	36	(33.0)
Baseline clinical condition		
Asymptomatic	75	(68.8)
Symptomatic	34	(31.2)
Associated STI		
Yes	16	(14.7)
No	93	(85.3)
Baseline CD4 cell count (cells/mm^3^)		
>350	75	(68.8)
≤350	34	(31.2)
Baseline viral load (copies/mL)		
<50	13	(11.9)
50–1000	7	(6.4)
>1000	89	(81.7)
Regimens based on DTG/TDF/3TC		
Yes	99	(90.8)
No	10	(9.2)

^†^Considering the year of 2017, the minimum wage = US$284,00; STI: sexually transmitted infection; DTG: dolutegravir; TDF: tenofovir disoproxil fumarate; 3TC: lamivudine.

**Table 2:** QoL dimensions measured at the start of treatment after 12 and 24 months of follow-up.

Quality of life dimensions (QoLD)	QoLD scores (T0)					QoLD scores (T1)					*p* value ^*∗*^	QoLD scores (T2)					*p* value ^*∗*^ ^*∗*^
	M	SD	Md	p25	p75	M	SD	Md	p25	p75		M	SD	Md	p25	p75	
Physical (D1)	15.4	3.5	16.0	14.0	18.0	16.4	3.1	17.0	15.0	19.0	≤0.001	16.7	2.7	18.0	15.0	19.0	≤0.001
Psychological (D2)	14.6	3.1	14.4	12.8	16.8	15.4	2.9	16.0	13.6	17.6	≤0.001	15.7	2.7	16.0	14.4	17.6	≤0.001
Level of independence (D3)	15.3	3.3	16.0	14.0	17.0	16.1	2.7	17.0	15.0	18.0	0.006	16.1	2.9	17.0	15.0	18.0	0.012
Social relationships (D4)	14.6	3.5	15.0	12.0	17.0	14.7	3.8	15.0	13.0	17.0	0.447	14.9	3.3	16.0	13.0	17.0	0.466
Environment (D5)	14.1	2.9	14.5	12.5	16.0	14.9	2.5	15.0	13.0	17.0	0.002	15.0	2.3	15.5	14.0	16.5	0.001
Spirituality/religion/personal beliefs (D6)	13.9	4.1	14.0	11.0	17.0	15.1	3.5	15.0	13.0	18.0	≤0.001	15.3	3.1	16.0	14.0	18.0	0.001
Total quality of life (TQoL)	14.6	2.5	14.8	13.3	16.3	15.4	2.3	15.7	13.8	17.3	≤0.001	15.6	2.1	15.9	14.5	17.2	≤0.001

M: mean; SD: standard deviation; Md: median; p25: percentile 25; p75: percentile 75; T0: beginning of the ART treatment; *T*1: 12 months follow-up; *T*2: 24 months follow-up; ^*∗*^Wilcoxon test, QoL scores T0, and QoL scores T1;  ^*∗*^ ^*∗*^Wilcoxon test, QoL scores T0, and QoL scores T2.

**Table 3:** Score factors of the way of coping scale measured after 12 and 24 months of follow-up.

Coping strategies	Factors scores 12 months					Factors scores 24 months					*p∗* value
	M	SD	Md	p25	p75	M	SD	Md	p25	p75	
Problem-focused strategies	3.8	0.7	3.8	3.3	4.3	3.7	0.6	3.8	3.3	4.2	0.844
Emotion-focused strategies	2.2	0.6	2.1	1.7	2.7	2.2	0.7	2.1	1.7	2.6	0.615
Search for religious practices/fanciful thinking	3.7	0.9	3.7	3.1	4.4	3.8	0.8	3.8	3.2	4.4	**0.003**
Search for social support	2.8	0.9	2.8	1.8	3.4	2.7	0.9	2.6	2.0	3.4	0.377

^*∗*^Wilcoxon test, factors scores 12 months, and factors scores 24 months. Bolded results are statistically significant; *p* < 0.05.

**Table 4:** Correlation between QoL domains and coping strategies.

Spearman correlation (*ρ*)	12 months				24 months			
	PFC	EFC	SRP	SSS	PFC	EFC	SRP	SSS
Physical (D1)	0.307 ^*∗*^	−0.226 ^*∗*^ ^*∗*^	−0.089 ^*∗*^ ^*∗*^ ^*∗*^	−0.058 ^*∗*^ ^*∗*^ ^*∗*^	0.043 ^*∗*^ ^*∗*^ ^*∗*^	−0.238 ^*∗*^ ^*∗*^	−0.066 ^*∗*^ ^*∗*^ ^*∗*^	−0.115 ^*∗*^ ^*∗*^ ^*∗*^
Psychological (D2)	0.328 ^*∗*^	−0.454 ^*∗*^	−0.050 ^*∗*^ ^*∗*^ ^*∗*^	0.050 ^*∗*^ ^*∗*^ ^*∗*^	0.260 ^*∗*^	−0.413 ^*∗*^	−0.037 ^*∗*^ ^*∗*^ ^*∗*^	0.002 ^*∗*^ ^*∗*^ ^*∗*^
Level of independence(D3)	0.220 ^*∗*^ ^*∗*^	−0.191 ^*∗*^ ^*∗*^ ^*∗*^	−0.221 ^*∗*^ ^*∗*^	0.004 ^*∗*^ ^*∗*^ ^*∗*^	0.065 ^*∗*^ ^*∗*^ ^*∗*^	−0.227 ^*∗*^ ^*∗*^	−0.191 ^*∗*^ ^*∗*^ ^*∗*^	0.027 ^*∗*^ ^*∗*^ ^*∗*^
Social relationships (D4)	0.471 ^*∗*^	−0.399 ^*∗*^	−0.215 ^*∗*^ ^*∗*^	0.266 ^*∗*^	0.304 ^*∗*^	−0.376 ^*∗*^	−0.031 ^*∗*^ ^*∗*^ ^*∗*^	0.248 ^*∗*^ ^*∗*^
Environment (D5)	0.383 ^*∗*^	−0.352 ^*∗*^	−0.200 ^*∗*^ ^*∗*^ ^*∗*^	0.163 ^*∗*^ ^*∗*^ ^*∗*^	0.320 ^*∗*^	−0.363 ^*∗*^	−0.096 ^*∗*^ ^*∗*^ ^*∗*^	0.179 ^*∗*^ ^*∗*^ ^*∗*^
Spirituality/religion/personal beliefs(D6)	0.095 ^*∗*^ ^*∗*^ ^*∗*^	−0.397 ^*∗*^	−0.090 ^*∗*^ ^*∗*^ ^*∗*^	−0.134 ^*∗*^ ^*∗*^ ^*∗*^	0.123 ^*∗*^ ^*∗*^ ^*∗*^	−0.392 ^*∗*^	−0.139 ^*∗*^ ^*∗*^ ^*∗*^	−0.077 ^*∗*^ ^*∗*^ ^*∗*^
Total quality of life (TQoL)	0.413 ^*∗*^	−0.438 ^*∗*^	−0.201 ^*∗*^ ^*∗*^ ^*∗*^	0.089 ^*∗*^ ^*∗*^ ^*∗*^	0.210 ^*∗*^ ^*∗*^	−0.475 ^*∗*^	−0.149 ^*∗*^ ^*∗*^ ^*∗*^	−0.061 ^*∗*^ ^*∗*^ ^*∗*^

^*∗*^*p* < 0.01,  ^*∗*^ ^*∗*^*p* < 0.05,  and  ^*∗*^ ^*∗*^ ^*∗*^*p* > 0.05; *n*: 103 (12 months); *n*: 99 (24months). QoL: quality of life; PFC: problem-focused coping; EFC: emotion-focused coping; SRP: search for religious practices/fanciful thinking; SSS: search for social support.

**Table 5:** Bivariate associations between QoL and coping strategies with adherence at 12 and 24 months of follow-up.

Time condition	12 months		*p* value	24 months		*p* value
	Adherents	Nonadherents		Adherents	Nonadherents	
	Md (p25–p75)	Md (p25–p75)		Md (p25–p75)	Md (p25–p75)	
Quality of life domains						
Physical	17.0(14.0–18,0)	18.0(16.5–19.5)	0.087 ^*∗*^ ^*∗*^	17.5(15.0–19.0)	18.0(16.0–19.0)	0.625 ^*∗*^ ^*∗*^
Psychological	16.0(13.6–17.6)	16.4(14.0–17.6)	0.510 ^*∗*^ ^*∗*^	16.0(14.4–17.6)	16.0(14.4–17.6)	0.604 ^*∗*^ ^*∗*^
Level of independence	16.0(14.0–18.0)	17(16.5–18.0)	0.069 ^*∗*^ ^*∗*^	17.0(14.5–18.0)	17.0(16.0–18.0)	0.285 ^*∗*^ ^*∗*^
Social relations	15.0(13.0–18.0)	14,5(12.0–17.0)	0.538 ^*∗*^ ^*∗*^	15.0(12.5–17.0)	17.0(14.0–18.0)	0.211 ^*∗*^ ^*∗*^
Environment	15.0(13.0–17.0)	15.0(13.0–16.8)	0.864 ^*∗*^ ^*∗*^	15.5(13.8–16.5)	15.0(14.0–17.0)	0.701 ^*∗*^ ^*∗*^
Spirituality	15.0(13.0–18.0)	17.0(13.5–19.5)	0.092 ^*∗*^ ^*∗*^	16.0(14.0–18.0)	15.0(13.0–17.0)	0.304 ^*∗*^ ^*∗*^
Total quality of life score	15.5(13.8–17.3)	16.8(14.3–17.5)	0.329 ^*∗*^ ^*∗*^	15.9(14.5–17.1)	15.8(14.8–17.8)	0.628 ^*∗*^ ^*∗*^
Coping strategies						
Problem-focused strategies	3.9(3.4–4.3)	3.6(3.1–4.3)	0.260 ^*∗*^	3.9(3.4–4.3)	3.8(2.9–4.1)	0.123 ^*∗*^
Emotion-focused strategies	2.1(1.7–2.7)	2.1(1.8–2.3)	0.950 ^*∗*^	2.1(1.7–2.7)	2.2(1.9–2.5)	0.852 ^*∗*^ ^*∗*^
Search for religious practices/fanciful thinking	3.7(3.1–4.4)	3.6(2.7–4.4)	0.596 ^*∗*^	3.8(3.3–4.5)	3.8(3.4–4.4)	0.943 ^*∗*^ ^*∗*^
Search for social support	2.8(2.0–3.6)	2.2(1.8–2.9)	**0.021** ^*∗*^ ^*∗*^	2.6(1.8–3.4)	2.6(2.2–3.2)	0.872 ^*∗*^ ^*∗*^

Bolded results are statistically significant *p* < 0.05;  ^*∗*^Student's *t*-test;  ^*∗*^ ^*∗*^Mann-Whitney *U*-test; QoL: quality of life; Md: median; p25–p75: interquartile range.

**Table 6:** Multivariate logistic regression factors associated with nonadherence in the study group.

QoL and CS scale score^†^	Odds ratio of nonadherence at follow-up model 8 (R_2N_ = 0.562) ^*∗*^			
	Md (p25–p75) score	aOR	(95% CI)	*p* value
Quality of life domains				
Physical	17 (15–19)	1.30	0.97–1.74	0.080
Spirituality	15(13–18)	1.41	1.06–1.88	**0.020**
Coping strategy				
Search for social support	2.8 (1.8–3.4)	0.240	0.09–0.69	**0.008**

Bolded results are statistically significant; *p* < 0.05. ^†^QoL: quality of life; CS: coping strategies, aOR: adjusted odds ratio;  ^*∗*^results from the final model of the multivariate logistic regression back stepwise model, showing only the factors remaining in the final model for the variables QoL and CS.

## Data Availability

The datasets used in this article are published on the Mendeley repository, https://data.mendeley.com/datasets/vvrwzztb2z/1.
